# Effects of bicycle infrastructure interventions on physical activity in the general population: rapid review

**DOI:** 10.1186/s12889-026-27110-z

**Published:** 2026-03-27

**Authors:** Lisa Stähler, Liane Günther, Leon Bäumer, Ferdinand Breuning, Simone Weyers, Claudia R. Pischke

**Affiliations:** Institute of Medical Sociology, Medical Faculty and University Hospital Düsseldorf, Heinrich Heine University Düsseldorf, 717, Moorenstr. 5, Düsseldorf, 40225 Germany

**Keywords:** Public health, Bicycle infrastructure, Intervention, Physical activity, Natural experiment

## Abstract

**Background:**

Despite the growing evidence on positive effects of built environment interventions, including bicycle infrastructure interventions, on physical activity, research methods employed in studies continue to be heterogeneous and the evidence on effective strategies is still lacking. The main objective of this rapid review was to systematically map the evidence regarding the effects of bicycle infrastructure interventions on physical activity in the general population of high-income countries.

**Methods:**

This rapid review adhered to Cochrane guidelines. A systematic search of PubMed was conducted using a predefined search strategy with the following key terms: bicycle infrastructure, multi- or single-strategic intervention and physical activity. Only English-language articles published between August 1st, 2013 and November 21st, 2025, were included. A pre-piloted data extraction spreadsheet was used to extract and summarize key information from the selected studies.

**Results:**

A total of 980 articles were screened for eligibility. Of these, 32 studies met the inclusion criteria and were included in the final analysis. The evidence revealed predominantly positive effects of bicycle infrastructure interventions on physical activity (*n* = 21). However, in 11 studies, no effects (*n* = 9) or decreased physical activity (*n* = 2) were found.

**Conclusion:**

These findings suggest that implementation of bicycle infrastructure may positively affect physical activity at the population level. Regional sociodemographic differences still have to be investigated to support current policy actions.

**Supplementary Information:**

The online version contains supplementary material available at 10.1186/s12889-026-27110-z.

## Background

Physical inactivity is one of the leading risk factors for non-communicable diseases (NCDs), such as diabetes type 2, various types of cancer, and cardiovascular diseases, as well as mental health disorders, such as depression and dementia. Insufficient physical activity (PA) is attributable for 7.2% of all-cause deaths, globally [1]. In the European Union (EU), one out of three adults do not meet the minimum requirements of 150 min moderate-intensity PA per week. If this threshold for PA was to be reached in the 27 member states of the EU, 11.5 million incident NCDs would be prevented by 2050 and health care costs of nearly 8 billion Euros per annum could be saved in EU member states [2]. In accordance with the United Nations’ Sustainable Development Goals (SDGs) for 2030, the aim is a 15% relative reduction of physical inactivity by 2030 worldwide [3]. From 2000 until 2022, the proportion of physically inactive individuals increased from 23% (900 million) to 31% (1.8 billion) worldwide. Should this trend continue, the target for PA will not be met. High-income Western countries are categorized as “off track”, although PA increased by almost 4% from 2000 to 2022, which is just below what is necessary to meet the 2030 target [4]. Other data confirm this upward trend suggesting that in 2019, 33% of the EU population spent more than 150 min per week on aerobic PA compared to 30% in 2014 [5].

The World Health Organization (WHO) has set out a framework in their Global Action Plan on PA 2018–2030 to tackle physical inactivity and its burden on four strategic policy areas: create active societies, create active environments, create active people and create active systems [3]. Some policy actions in the EU were aimed at building environments that enable regular PA of citizens. For example, in Luxembourg, roads were closed throughout the city which led to an additional 600 km of cycling opportunities during the “Luxembourg Bicycle Summer” for 30 days [2]. In Germany, the expansion of the national cycle paths (“Cycling Network Germany”) in order to create safe, attractive, and seamless cycling infrastructure integrated into the European cycle route “Iron Curtain”, is currently being financed by the federal government [6].

Emerging evidence reveals that infrastructural bicycle interventions have a positive effect on PA, but study designs, as well as research methods, vary greatly and the causal pathways between infrastructural changes and behavioral changes towards a more active lifestyle have to be validated. Grunseit et al. [7] conducted a natural experiment to examine use of an entirely motorized traffic-free walking and cycling loop trail in the northern suburbs of Sydney. Their results are based on time series analysis of visual and electronic count data before and after the completion of the trail and show that the usage of the trail by cyclists increased threefold, with 7% more children who utilized the trail compared to baseline. In a controlled natural experiment, Goodman and colleagues [8] compared census data from 18 “intervention towns” across England with census data from matched comparison towns. Cycling initiatives were town-adjusted and ranged from infrastructural provisions (i.e., on road cycle lanes, cycle parking spaces) to promotional activities (cycle trainings i.e., in schools). Over the course of the decennial English census from 2001 to 2011, cycling increased significantly in the intervention compared to the comparison towns. Hirsch et al. [9] found that proximity to new transport infrastructure is a key determinant to increase bicycle commuting, preferably more than a well-equipped cycling environment [10]. In contrast, Heinen and colleagues [11] were unable to show that proximity to a newly built busway with cycling paths was associated with marked changes in travel behavior but could demonstrate rather stable, non-random active travel patterns.

The main objective of this rapid review is to systematically map existing evidence on bicycle infrastructure interventions and their impact on PA in the general population. This review adds to the existing evidence by comparing effects of single- and multi-strategic infrastructure interventions and by assessing the role of participatory design approaches in existing studies. We want to answer the following main research question: (1) What are the effects of interventions aimed at improving bicycle infrastructure on PA in the general population? In addition, the following secondary research questions are examined: (2) Which intervention components were employed in these interventions? (3) Were participatory design approaches used? (4) Which outcome measures and study designs were used in previous intervention studies? (5) How was reach, adoption, implementation, and maintenance assessed in previous intervention studies?

## Methods

The review was registered at PROSPERO (reference number CRD42023456683 [12]), and conducted in accordance with the Cochrane Rapid Review Methods recommendations [13].

### Search methods

As outlined in the guidelines, a stepwise approach was employed. On August 1st, 2023, a search was conducted in the PubMed database to identify eligible studies. To ensure the inclusion of more recent evidence, the search was updated on November 21st, 2025, using the same search strategy and eligibility criteria. Additionally, snowballing was applied by reviewing the reference lists of the systematic reviews identified in the PubMed search. The search terms and filters applied are shown in detail in Supplementary Material A. Three main search concepts were combined: 1) PA, representing outcomes related to movement; 2) bicycle infrastructure, representing changes to the built environment that support cycling; and 3) intervention strategies, representing both single and multi-strategic approaches to promote cycling.

### Eligibility criteria

Studies published in English in the last 10 years were considered eligible, if they met the following criteria: (1) employed a quantitative (randomized controlled trials, intervention studies, pragmatic trials, observational studies, natural experiments) or qualitative study design, (2) involved multi-strategic or single-component interventions aimed at improving bicycle infrastructure, (3) measured PA, including cycling as the primary outcome, (4) measured subjective well-being, quality of life and other health-related outcomes as secondary outcomes, (5) were conducted in urban areas in high-income countries.

### Screening

To facilitate the title and abstract screening process, the online screening tool Rayyan [14] was used. Following the import and de-duplication of retrieved records, two reviewers (initial search: LS, LB; updated search: LS, SW) independently screened each title and abstract for potentially eligible studies. Full-texts were then retrieved for further assessment and screened by the same two independent reviewers (initial search: LS, LB; updated search: LS, SW). Conflicts were resolved by a third reviewer (initial search: SW; updated search: CRP) in both screening processes.

### Data extraction

Two reviewers (initial search: LS, LB; updated search: LS) jointly extracted data from the included studies using a previously piloted data extraction form. The data were extracted according to the following categories: study characteristics (e.g., author, year of publication, study design, country), participant characteristics (e.g., age, gender), intervention details (e.g., intervention components, intervention duration, intervention groups), outcomes (e.g., PA, health-related outcomes) and measurement instruments. The extracted data were then analyzed to address the research questions. Any questions or discrepancies that arose during the data extraction and analysis process were resolved through discussion in the research team.

### Risk of bias assessment

To assess the internal validity of the included studies, the ROBINS-I tool for non-randomized studies of interventions [15] was employed. Signaling questions on six out of the seven Risk of Bias (RoB) domains 1) confounding, 2) selection of participants into the study, 3) classification of interventions, 4) missing data, 5) measurement of outcomes, and 6) selection of reported results were answered jointly by two reviewers (LG, CRP for original and updated results). Based on the number of answers marked green (potential for low RoB) or red (potential for serious RoB), each domain was rated by the two reviewers in terms of revealing a low RoB, a moderate RoB, a serious RoB or a critical RoB. Lastly, domain specific ratings were summed up to an overall RoB judgement for each study. Although the tool is specifically designed to assess RoB in non-randomized studies comparing the health effects of two or more interventions, its reliability and applicability to natural experiments have been criticized due to a lack of consensus, particularly regarding domains 2) and 4) [16]. The applicability of both domains was critically appraised in the research team resulting in the inclusion of domain 2) only and exclusion of domain 4) (deviations from intended interventions) as it was deemed unsuitable in the context of natural experiments.

## Results

### Search outcomes

**Fig. 1:**
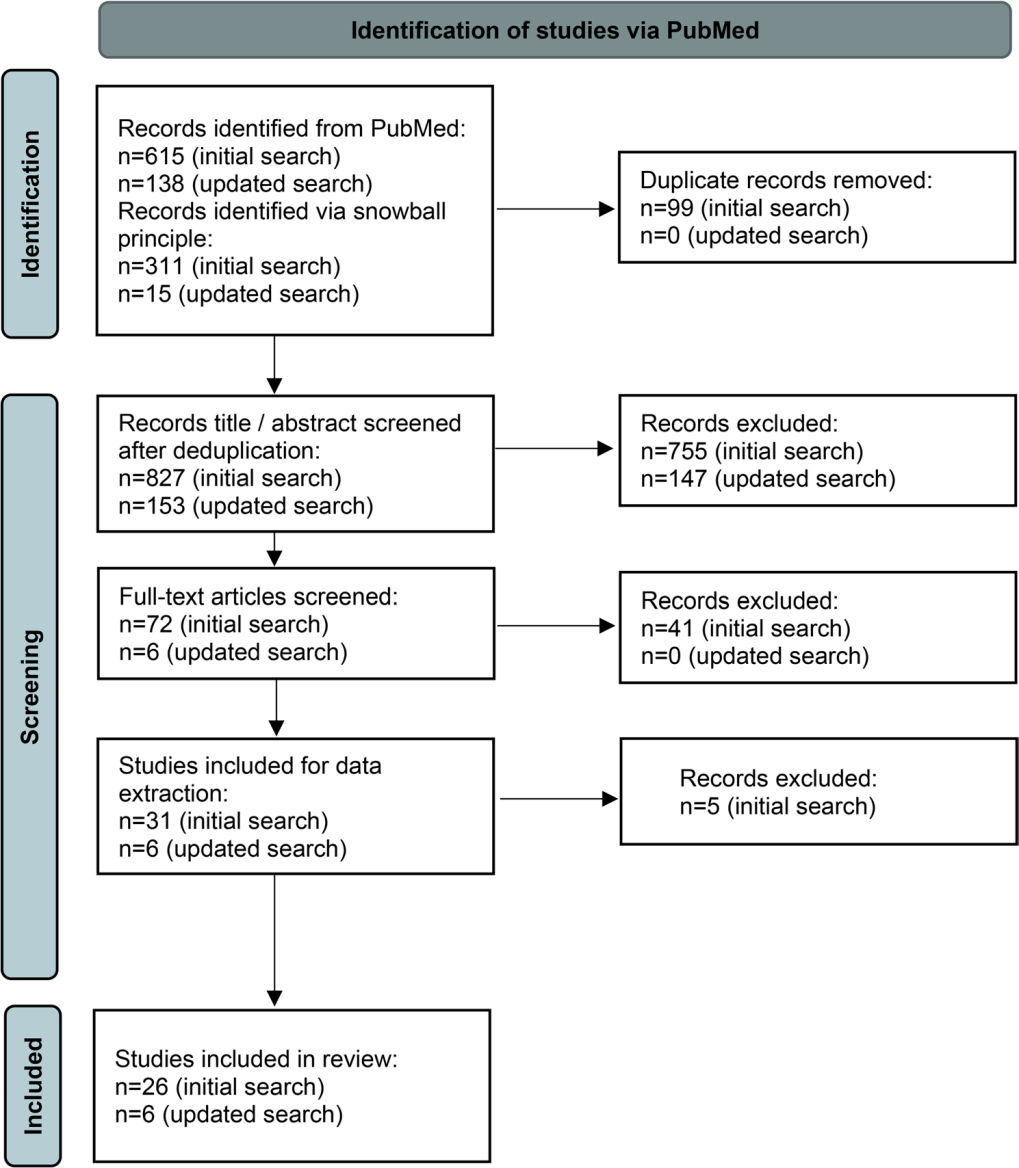
Flowchart

Figure 1 illustrates the study selection process. A total of 753 studies (initial search: *n* = 615, updated search: *n* = 138) were identified during the search of the PubMed database and imported into the Rayyan screening tool [14]. An additional 326 studies (initial search: *n* = 311, updated search: *n* = 15) were identified using the snowball method, which resulted in 1,079 potentially relevant studies. Across both searches, 99 duplicates were removed (all from the initial search), leaving a total of 980 records included in the title and abstract screening. As a result, 78 articles (initial search: *n* = 72, updated search: *n* = 6) were retrieved for full-text screening, of which 37 (initial search: *n* = 31, updated search: *n* = 6) met the eligibility criteria and were included in the data extraction. During data extraction, five additional studies from the initial search were excluded, yielding a final total of 32 studies included in this review. In Supplementary Material B, references and characteristics of the included studies are provided. It should be noted that multiple studies investigated the same interventions. Specifically, the “Connect2” [17–19],“Cambridgeshire Guided Busway” [11, 20, 21], “Cycling Demonstration Towns (CDTs) and Cycling Cities and Towns (CCTs)” [8, 22], and “mini-Holland” [23, 24] interventions, each of which were the focus of several independent analyses.

### General study characteristics

#### Publication date and country

The included studies were published between 2013 and 2023 and were conducted in eight different countries: the United States of America (USA) [9, 25–30], Canada [31, 32], Australia [7, 33–36], New Zealand [37], Singapore [38], the United Kingdom (UK) [8, 10, 11, 17–24, 39], France [40], Finland [41], the Netherlands [42], and Denmark [43]. The majority of studies (*n* = 18) were conducted in the USA and the UK.

#### Population

All of the included studies focused on participants from the general population. Three studies noted an underrepresentation of younger, non-white and unemployed participants [23, 24, 33], one study specifically examined employees of a medium-sized company [41], one included residents from a disadvantaged area (predominantly residents with African American ethnicity, high crime rates, low employment rates [25]) and one considered different levels of socio-economic deprivation [8].

#### Age

Participant age varied across the included studies. Nineteen studies included adults 16 + years [8, 11, 20–24, 39], 18 + years ([7] survey, [17–19, 26, 27, 32–34, 36, 42]), inclusion of “adults” without age specification [29, 35], five studies included participants of all ages ([7] counters, [28, 31], [37] NZTS survey, [38, 41]), one study included participants 10 + years ([37] ACTIVE survey), and six studies did not specify participant age [9, 10, 25, 30, 40, 43].

### Risk of bias

The overall RoB assessments of the included studies (*n* = 32) from both searches revealed that 18 studies [7–10, 21, 23–26, 28, 30, 31, 34–36, 39, 40, 42] were considered having a low RoB and six as having a moderate RoB [11, 17–20, 33] (see Table 1). In 11 studies [7, 25, 27–29, 33, 35, 36, 38, 41, 43], a combination of objective and subjective measurement methods was used to assess the outcomes of interest. Observational counts of cyclists and pedestrians done by members of the respective research team were rated as subjective measurement (e.g., [10, 30, 31]). The two measurement methods were judged separately for RoB domains four to six which led to a different overall RoB rating (low or moderate) for two studies [29, 41]. Another six studies [22, 27, 32, 37, 38, 43] were rated as “no information”.

In six studies, the moderate RoB was due to missing data that exceeded a 10% threshold and led to exclusion from data analysis. Five of the studies with an overall moderate RoB rating [11, 17, 18, 20, 33] were potentially biased because survey data were either self-reported by participants or provided by unblinded assessors who were aware of the intervention. Furthermore, in four of these studies [17, 18, 33, 41], different intervention periods for participants posed a risk of bias, mostly caused by delays in the infrastructural constructions.

In the studies by Aittasalo et al. [41] and Dill et al. [29], the overall RoB was rated differently. They used surveys to assess active transportation and enriched them with data from accelerometry and Global Positioning System (GPS). Evaluating accelerometer and GPS as measurement tools resulted in a low RoB judgement for both studies compared to the surveys, which may have been influenced by knowledge of the intervention and therefore were rated as moderate RoB. In other studies, the combination of objective and subjective measurement methods did not affect the overall RoB rating.

The remaining six studies [24, 27, 38, 40, 42, 43] lacked information on the proportion of missing data and how missing data were dealt with in the analysis. Therefore, a final RoB judgement could not be reached.

**Table 1:**
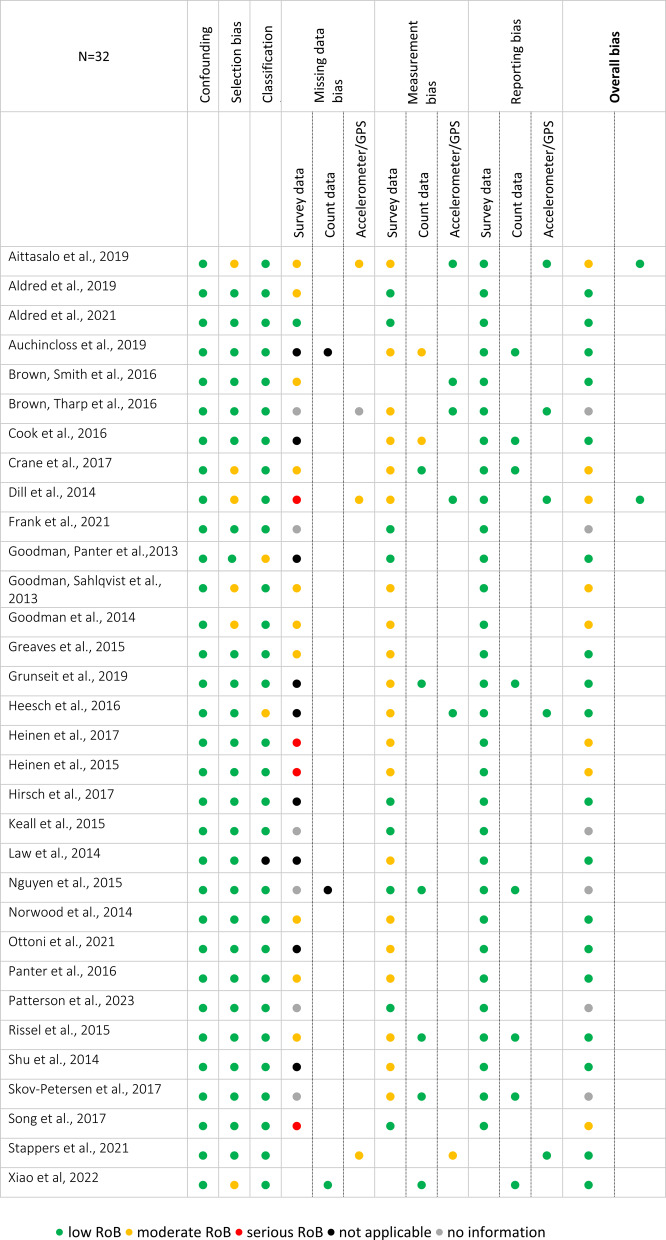
Risk of Bias Scores of the included studies

### Main research question: what are the effects of interventions aimed at improving bicycle infrastructure on physical activity in the general population?

In total, 21 studies reported a positive effect on PA, either in cycling or walking, due to the implementation of new infrastructures or segmental improvements of existing routes. Of these 21 studies, seven reported an increase in PA over time [7, 10, 17, 19, 28, 30, 43], and seven studies [8, 22–24, 35–37] revealed increased cycling when comparing the proportion of cyclists pre-/post-infrastructural intervention to a control group or alternative cycle routes. The remaining seven studies [9, 18, 20, 21, 26, 32, 33] compared effects of infrastructural changes on residents living in differing proximity to the site finding that resident living closer benefited the most. Furthermore, in nine studies [11, 25, 27, 31, 34, 38, 40–42], no overall effects on PA after the implementation of bicycle infrastructure were observed and two studies [29, 39] revealed a decline in PA. In the following sections, we focus on presenting the results of *n* = 21 studies reporting significant positive changes, detailed information on the remaining studies can be found in Supplementary Material B.

Cook et al. [28] found a 6% increase in the proportion of cyclists after the completion of a bicycle and pedestrian bridge that connected two parts of a trail, whereas the proportion of runners shrunk by 6%. The average trip duration across all transportation modes increased by 4 min, with 6 min average increase spent by cyclists. Grunseit et al. [7] found that mean automatic bike and pedestrian counts were higher post-completion of an infrastructural intervention. However, the majority of infrastructure users were not meeting the PA guidelines but were more likely to report increased PA at 10.5 weeks follow-up compared to users meeting PA guidelines. Skov-Petersen et al. [43] observed increases in the number of cyclists during different periods of the day on two renewed infrastructure sites, a cycle greenway and a cycle highway. Particularly after the reopening of the cycle highway in 2012, the cyclist volume rose during all periods of the day on the cycle highway. For example, there was a 61% increase from 126 (95% Confidence Interval [CI] 122, 130) to 203 bicycles/hour (95% CI 195, 210) on weekdays during daylight rush hour. On the cycle greenway, there was a 71% increase from 14 (95% CI 8, 20) to 24 bicycles/hour (95% CI 12, 37) during dark rush hour during weekdays. Over the course of one year, the number of individuals that changed their cycle route (49–61%) or introduced cycling as a mode of transport to their lives (4–6%) also increased. In another study, Law et al. [10] found an increase in cyclist movement patterns between 2003 and 2012, however, a regression analysis revealed that despite the changes in cycling infrastructure, 55% of the variation in cyclist movement patterns in 2012 relied on earlier cyclist movement patterns in 2003. Cyclists preferred more direct and continuous routes compared to routes with better infrastructure but less direct paths at both times. Shu et al. [30] noted an average 37% increase in pedestrians after an infrastructure implementation, across morning, afternoon, evening sessions on weekdays and weekends (all *p*<.05, except weekday mornings), but no changes in cyclist volume. Song et al. [19] observed a shift from car use to active travel (walking and cycling) in 21–25% of participants, but also an inverse shift from active travel to car use for 20–23%. Actual use of the infrastructure was associated with a shift towards active travel, both at one- and two-year follow-up. However, at two-year follow-up, walking decreased (mean time=-13.30, *p*=.021). Goodman, Sahlqvist et al. [17] reported a 6% increase (32–38%, *r*=.62) in use of new walking and cycling paths from one- to two-year-follow-up, with walking for recreation being the most reported reason (84–85%, *r*=.60), followed by cycling for recreation (37–39%, *r*=.65). The infrastructure was used less for transportation purposes, both on foot (35 − 32%, *r*=.51) and by bike (17–18%, *r*=.60).

Aldred et al. [23] found that the part of the intervention group that was most exposed to substantial changes in local walking and cycling infrastructure, was 7.2% more likely to report cycling in the past week compared to the control group, and experienced a 41-minute increase in active travel time (*p*=.02) at one-year follow-up. These effects persisted beyond the first year. In a later study, Aldred et al. [24] observed 44-minute (*p*<.05) and 42-minute increases in active travel time at two-year and three-year follow-ups, respectively. Heesch et al. [35] found an increase in monthly GPS cycle counts on a new segmental bike path linking Brisbane’s south to the city centre compared to no change on alternative routes. Rissel et al. [36] found a greater use of a new bi-directional bicycle path in the intervention compared to the control group at follow-up (IG = 23.8%, CG = 7%, *p*=.001). However, no change in frequency of past week cycling was observed in either of the groups: Weekly cycle frequency remained higher in the intervention group (IG = 29.2–25.8%, CG = 22.4–23.2%, *p*=.04), and remained stable over time. Goodman, Panter et al. [8] detected an increase in cycling and walking to work in intervention towns, when analysing the decennial English census from 2001 to 2011. The absolute effect size for cycling to work was + 0.69 (95% CI 0.60, 0.77) and the relative effect size was + 1.09 (95% CI 1.07, 1.11) when compared to comparison towns. Further, the prevalence of walking to work increased by + 1.71 (95% CI 1.62, 1.81) percentage points in the intervention cities. Extending these findings using the same census data, Patterson et al. [22] detected increases in cycle commuting prevalence (Adjusted Odds Ratio [AOR]=1.56, 95% CI 1.16, 2.10), and in cycle commuting uptake (AOR=2.13, 95% CI 1.56, 2.91) in the intervention towns compared to control towns, observed only among women. Lastly, Keall et al. [37] observed a 37% increase in the odds of active travel trips in two intervention towns that received new bicycle infrastructure, active travel campaigns and bicycle education over two years, compared to control towns without comparable funding (Odds Ratio [OR]=1.37, 95% CI 1.08, 1.73).

In seven studies that identified positive effects on PA, proximity to the intervention site was associated with greater engagement in PA. Goodman, Sahlqvist et al. [18] found that living nearer the intervention site predicted changes in PA-levels at two-year follow-up. Every kilometre closer to the infrastructure was associated with 15.3 min more walking and cycling (95% CI 6.5, 24.2) and 12.5 more minutes of total PA (95% CI 1.9, 23.1) per week. The effect was moderated by car ownership, revealing that car owners spent fewer minutes in walking and cycling per week (10.2 per km closer to intervention site, 95% CI 0.3, 20.1) compared to those who did not have access to a car (46.8 per km closer to intervention site; 95% CI 21.6, 72.1, interaction *p*=.007). Panter et al. [21] found that a 4 km distance in comparison to a 9 km distance to the infrastructure was associated with a 34% higher likelihood to increase cycle commuting time (Relative Risk Ratio [RRR]=1.34, 95% CI 1.03, 1.76, *p*<.05). Furthermore, the authors reported more total time spent in cycling (RRR=1.32, 95% CI 1.04, 1.68, *p*<.05) and more overall active commuting time among those who had been the least active commuters at baseline (RRR=1.76, 95% CI 1.16, 2.67). Brown, Smith et al. [26] found that residents living ≤ 800 m from the intervention site were more likely to take transit-related active transportation trips (all *p*<.04) and walking trips (all *p*<.001) at follow-up, compared to residents living 801–2000 m from the intervention site both at baseline and at follow-up. The proportion of cyclists was lower, in general, but differed between intervention group at follow-up compared to the reference group at baseline (10% vs. 5%). Crane et al. [33] noted that individuals living in the intervention area were four times more likely to use the cycleway and a strong correlation was observed between weekly cycling and cycleway use. The intervention group also showed a higher frequency of cycling compared to the control group and participants residing between 1 and 2.99 km compared to participants residing closer (< 1 km) or farther (> 3 km) from the cycleway demonstrated an increase in minutes of cycling. Heinen et al. [20] observed that commuters living closer (4 km) compared to commuters living further (9 km) from the intervention site were nearly twice as likely to report an increase (> 30%) in the share of trips involving active travel (RRR=1.80, 95% CI 1.27, 2.55), and a decrease (> 30%) in the share of trips made by car by > 30% (RRR=2.09, 95% CI 1.35, 3.21). However, no increases in number of commute trips or in commute distance were seen. Hirsch et al. [9] reported a 2.3% increase in commuting by bicycle over a decade. Proximity to and crossing of a new trail system were positively associated with increases in bicycle commuting (*p*<.01), even after adjusting for covariates, such as socio-demographic variables (e.g., population density). Lastly, Frank et al. [32] observed that proximity to a retrofitted 2 km greenway was associated with higher cycling trip frequency: Participants living within a 300 m radius of the intervention site compared to participants living further away showed a 252% increase in cycling trips (Incidence Rate Ratio [IRR]=3.52, 95% CI 1.54, 8.03, *p*=.003).

### Secondary research questions

#### Which intervention components were employed in these interventions?

##### Intervention components

All included studies examined the effects of bicycle infrastructure interventions which included the construction of new trails and trail segments, and the implementation of improvements of existing infrastructure. Twenty studies [7, 8, 10, 11, 20–27, 30–33, 37, 39, 41, 42] employed a multi-strategy approach that in addition to bicycle infrastructure encompassed other components, such as public transportation infrastructure, environmental adjustments (e.g., implementation of more greenery), or social-behavioral intervention components. For instance, Goodman, Panter et al. [8] evaluated a multi-strategy intervention across 18 towns, which, in addition to bicycle infrastructure, included school bicycle trainings, maps for backstreet routes to schools, cycling taster sessions, on-site cycle repairs for employees, training in cycle maintenance, and cycling festivals. See Supplementary Materials B and C for an overview of intervention components evaluated in the included studies.

##### Intervention groups

The number of intervention and control groups in the included studies varied: The majority of studies (*n* = 15) included one intervention group and no control group (1 IG, 0 CG) ([7, 9–11, 17–21, 27, 28, 30, 31, 38], [41] study part 1), whereas 14 studies included one intervention and one control group (1 IG, 1 CG) ([23–26, 29, 32–34, 36, 37, 39, 40], [41] study part 2, [43]), and in four studies, multiple intervention or control groups were compared [8, 22, 35, 42].

Intervention groups included on-site users and citizens living in or in proximity to the intervention area. The distances to the intervention area differed between studies. For example, Brown, Smith et al. [26] categorized participants into the intervention group based on an 800 m radius, whereas the control group was defined as living within an 801–2000 m radius. In contrast, Rissel et al. [36] included participants living up until a 2500 m radius in the intervention group. Control groups were areas comparable in composition to the intervention areas, but situated at a greater distance from the intervention sites. In the case of Goodman, Panter et al. [8], who examined interventions in several cities, control groups were comparison cities of a similar size, but not targeted by the intervention. It should be noted that the control areas did not necessarily lack bicycle infrastructure, but were selected based on their distance to the new infrastructure.

##### Intervention duration

Most studies (*n* = 17) had a one-to-two-year interval between baseline and follow-up measurements ([7] Ecounter, [17–19, 23, 26–34, 36–38, 41–43]). Apart from that, three studies had a duration of less than one year between baseline and follow-up ([7] visual counts, [35] GPS, [40]), and ten studies had a duration of three years [11, 20, 21, 25, 39, 43], or more ([8–10], [35] field observation, intercept survey).

It should be noted that the time interval between baseline and follow-up assessments did not necessarily correspond to the duration of the intervention implementation, i.e., the time between the completion of the intervention and the last follow-up assessment. For example, in the study by Shu et al. [30], the baseline and follow-up assessments were two years apart, yet, the time between the completion of the intervention and follow-up assessment was only two months.

#### Were participatory design approaches used?

In only two studies, a participatory design approach was used: In Ottoni et al. [31], prior to the implementation of the intervention, 4,000 residents were invited to provide feedback on the development of the new intervention through a public consultation process. In Goodman, Sahlqvist et al. [18], local authorities or community groups could apply for participation in intervention projects.

#### Which outcome measures and study designs were used in previous intervention studies?

##### Measurement instruments

The changes in PA were either subjectively or objectively measured. Subjective measurement instruments included travel diaries, surveys and manual counts. Objective measures included GPS trackers, accelerometers, automated counters, the System for Observing Play and Recreation in Communities (SOPARC), and the Path Environmental Audit Tool (PEAT). Eighteen studies used subjective instruments only [8–11, 17–24, 30–32, 34, 37, 39], while two studies used objective instruments only [26, 40], and 12 studies used a combination of both [7, 25, 27–29, 33, 35, 36, 38, 41–43].

##### Study design

All 32 studies were natural experiments with a pre-post design that evaluated government funded interventions. In the majority of the studies (*n *= 28), data were collected by the research team, while in four studies, census data were used as the primary [8, 9, 22] or secondary (sociodemographic information only) data source [25]. Paralleling a natural experiment, one study included an aggregate data model [10], and one study conducted a randomized controlled trial [41].

#### How was reach, adoption, implementation, and maintenance assessed in previous intervention studies?

Of the 32 included studies, only Aittasalo et al. [41] conducted a process evaluation. The intervention was rolled out in two phases: phase 1 involved improvements on main and connecting walking and bicycling paths from 2014 to 2016. Phase 2 involved the distribution of social-behavioral work books for promoting active commuting to work (ACW) along with educational material in companies from Fall 2016 to Spring 2017. Regarding reach, 11 (30%) of the 37 companies initially approached and 900 (49.4%) of 1,823 employees participated in phase 1. In phase 2, 16 (36%) companies (including 11 from phase 1 and 5 additional approached companies in phase 2), and 630 (51.3%) of 1,228 employees participated. Regarding the implementation of the intervention, delays in infrastructural improvements in phase 1 reduced the intervention exposure time from six to two months. In phase 2, social and behavioral action plans were developed and implemented as planned. Mixed results were obtained on employees’ recognition of the implemented strategies. In phase 1, 46% of participants reported preparing to alter their ACW (e.g., by repairing or purchasing bicycles, seeking suitable routes), but most participants (99% for walking and 95% for bicycling outcomes) stated that the improvements had no impact on their ACW. Similarly, 55% of phase 2 employees reported knowing of the social and behavioral strategies to promote ACW, but only 26% reported participating in them.

## Discussion

This rapid review systematically summarized the published literature from 2013 onwards and provided an update of the evidence on the impact of bicycle infrastructure interventions on PA in the general population. Addressing research question 1, we found predominantly positive effects of infrastructural interventions on PA in the studies included in this rapid review. Prevailing was the increase in cyclist volume on the new infrastructures followed by an increase in the number of pedestrians. Further, more active travel trips and increases in the time spent on active travel were recorded. One moderator for the use of such infrastructures and the associated increased PA-levels appears to be the proximity to the new or improved infrastructure which is in line with the results by Stappers et al. [44] who found a link between proximity and increases in cycling as well. It remains to be further investigated whether proximity plays a role, in general, or when using the infrastructures for activities of daily life, such as commuting to work which was reported in a marginal number of included studies. A handful of studies failed to demonstrate a quantifiable effect on PA, and in two studies an undesirable decrease in PA was found at follow-up. Similar mixed results were reported in the work of Smith et al. [45] on PA and active transportation in adults and children. Mayne et al. [46] reported little impact on total PA in their review but found consistent positive process outcomes in line with the intended interventions (e.g., increases in cycling after bike infrastructure changes).

Pertaining to research question 2, we found that the majority of the employed interventions were multi-strategic, including combinations of changes in the built environment beyond improvements of the cycling infrastructure, such as, a new busway, greening of the area or improvement of lighting conditions. In some interventions, social-behavioral strategies were added to foster PA, for example information brochures or bicycle trainings. In the single component interventions, mainly a bike/walking trail or bridge was either constructed or renewed. We cannot confirm the conclusions drawn by Stappers and colleagues [44] stating that single interventions had a positive impact on bicycling whereas comprehensive environmental changes mainly were ineffective. We found that positive, as well as negative/non-significant outcomes, were observed after both multiple- and single-component interventions.

Regarding research question 3, we were able to identify only two studies employing participatory design approaches and one study conducting a process evaluation. This might be due to the observational nature of all included studies, which do not aim for ideal research conditions to guarantee a high internal validity rather than examining policy actions, or programs under real life conditions [47]. It is regrettable that we did not find more studies employing participatory design approaches, as they can strengthen ownership among stakeholders and citizens, improve contextual fit, and support long-term sustainability [48, 49]. For example, in four major German cities, the grassroot movement “Radentscheid” (Engl. Cycle ruling) led to the institutionalization and inclusion of cycling on political agendas [50] demonstrating the potential impact of participatory approaches in the long term.

Pertaining to research question 4, we found that the heterogeneity of the included studies in methodology, intervention typologies and characteristics, and outcome measurements was substantial. For example, we found time intervals from under six months up to ten years between baseline and follow-up; or a study that analyzed census data on cycling and walking to work from 18 interventions towns versus a study that counted users of a partially finished greenway for 24 h per observation. This made it difficult to compare the studies with each other and draw general conclusions or to establish causal relationships between the environmental interventions and behavioral changes in PA parameters. Unlike other authors, we found that objective measurement tools, such as trackers or accelerometers, were more commonly incorporated [44, 46, 51] and mixed methods were used to assess the outcomes of interest. This is not surprising, as technology has evolved and complementary assessments are promising to gain insights into movement, PA, and sedentary behaviors under real life conditions and across different populations [52].

This has arguably influenced our overall RoB ratings which were mostly low and, in some cases, moderate, as shown in Table 1. Our domain specific ratings suggested that count and accelerometer data were considered less prone to bias compared to survey data. The handling of missing data across all studies was problematic because of small retention rates at follow-up on the one hand, as well as a lack of reporting of missing data and low comprehensibility of cases/ data sets when the study population was purely counted per observation.

This study had several limitations and strengths that should be considered when interpreting the findings. First, although our steps were in line with the Cochrane Rapid Review methods recommendations [13] to limit the literature search to one database and to not include grey literature, we may not have captured all relevant studies. Second, the RoB quality assessments have to be interpreted with caution due to limitations of the ROBINS 1-tool. In line with Thomson et al. [16], domain four was not applicable to the included natural experiments in which subjects are typically not clearly assigned to an intervention. We also acknowledge that domain five was difficult to apply whenever different pools of individuals at two or more measurement points were randomly counted or surveyed while using the intervention site. Furthermore, an overall judgement with a “no information” rating appears overly strict as it mainly refers to missing information in domain five regarding the distribution of drop-outs over groups and sensitivity analysis.

One of the strengths of this review is its systematic approach to identify, review, extract, evaluate and summarize eligible literature according to the Cochrane Rapid Review methods recommendations [13]. Another strength is the additional inclusion of a second and third reviewer. Literature screening and the risk of bias assessment were executed by two reviewers and inconsistencies in judgements were discussed with a third reviewer.

## Conclusions

To conclude, this rapid review identified evidence suggesting a benefit of infrastructure interventions with regard to various types of PA. A very small number of studies were conducted in Europe and it remains to be investigated whether the above-mentioned results of infrastructural interventions on PA, including cycling and walking or active transport, are transferable to somewhat different geographic conditions in Europe as well as socio-demographic compositions of European populations. The included studies predominantly examined the general population and did not look at socio-economically disadvantaged populations or areas, and there is no evidence on (potentially differential) effects of building or extending infrastructure in Europe. This aspect warrants further investigation. It will be essential in the future, however, to inform current policy makers with valid data to support them in decision making regarding the creation of PA promoting and sustainable environments, while facing upcoming climate and environmental challenges. Thus, the construction of new bicycle infrastructure should be accompanied by studies employing intervention and control sites, involving triangulation of assessments of PA, and measuring potential changes of PA at the population level over longer (>1 year) follow-ups to be able to track whether the population progressively meets the targets of the Global Action Plan.

## Supplementary Information

Supplementary Material 1.

## Data Availability

Any datasets other than those provided in this manuscript or its supplementary materials are available from the corresponding author on reasonable request.

## Abbreviations

NCD: Non-Communicable Disease
PA: Physical Activity
EU: European Union
SDGS: Sustainable Development Goals
WHO: World Health Organisation
ROB: Risk of Bias Assessment
CDT: Cycling Demonstration Towns
CCT: Cycling Cities and Towns
USA: United States of America
UK: United Kingdom
GPS: Global Positioning System
CI: Confidence Interval
AOR: Adjusted Odds Ratio
OR: Odds Ratio
RRR: Relative Risk Ratio
IRR: Incidence Rate Ratio
IG: Intervention Group
CG: Control Group
SOPARC: System for Observing Play and Recreation in Communities
PEAT: Path Environmental Audit Tool
ACW: Active Commuting to Work
