# Supplementary material for: Effects of bicycle infrastructure interventions on physical activity in the general population: rapid review
Source: BMC Public Health. 2026 Mar 27;26:1169. doi: 10.1186/s12889-026-27110-z (PMC13063923; doi:10.1186/s12889-026-27110-z)
Supplement: Supplementary file 1 — Supplementary Material 1. [file 12889_2026_27110_MOESM1_ESM.docx]

Supplementary Materials

Supplementary Material A: Pubmed Search Strategy

#1 "physical activ*"[tiab] OR "motor activ*"[mh] OR "motor activ*"[tiab] OR exercise*[mh] OR exercise*[tiab] OR ("physical education"[tiab] AND training[tiab]) OR sport*[mh] OR sport*[tiab] OR "physical fitness"[mh] OR "physical fitness"[tiab] walk*[tiab] OR "nordic walk*"[tiab] OR jogg*[tiab] OR swim*[tiab] OR "weight lift*"[tiab] OR danc*[tiab] OR aerobic*[tiab] OR "circuit train*"[tiab] OR "Circuit-Based Exercise*"[tiab] OR "weight train*"[tiab] OR "cross train*"[tiab] OR "endurance train*"[tiab] OR "resistance train*"[tiab] OR „Exercise test*"[tiab] OR „Exercise therap*"[mh] OR „Physical Exert*"[mh] OR „Physical Exert*"[tiab] OR "Human Physical Conditioning"[tiab] OR "Physical Endurance"[tiab] OR „Acute Exercise*"[tiab] OR "Isometric Exercise*"[tiab] OR "Aerobic Exercise*"[tiab] OR "Exercise Training*"[tiab] OR "Physical Education*"[tiab] OR Athletic*[tiab] OR "Strength Train*"[tiab] OR "Weight-Lifting Exercise Program*"[tiab] OR "Weight-Bearing Strengthening Program*"[tiab] OR "Weight-Bearing Exercise Program*"[tiab] OR "Physical Effort*"[tiab] OR "Rehabilitation Exercise*"[tiab] OR "physical activity behavior*"[tiab] OR "Leisure-time physical activ*"[tiab] OR "Moderate-intensity physical activ*"[tiab] OR "Vigorous-intensity physical activ*"[tiab] OR "physical activity level*"[tiab] OR "patterns of physical activ*"[tiab] OR "Physical performance*"[tiab] OR "high-intensity interval training*"[tiab] OR HIIT[tiab] OR "transport related activ*"[tiab]

#2 “Bicycle lane*” [tiab] OR “Bicycle network*” [tiab] OR “Bicycle infrastructure*" [tiab] OR “bicycle facilities*” [tiab] OR “Cycling infrastructure*” [tiab] OR “Bike way*” [tiab] OR “Bike path*” [tiab] OR “Cycling path*” [tiab] OR “Bike parking” [tiab] OR “Urban form*” [tiab] OR “Urban planning” [tiab] OR “City planning” [MESH] OR “city planning” [tiab] OR “Urban polic*” [tiab] OR “Built environment*” [MESH] OR “Built environment*” [tiab] OR “Bicycle boulevard*”[tiab] OR “Street connectivit*” [tiab] OR cycleway* [tiab] OR “active transport*”[tiab]

#3 “multi strategic intervention*”[tiab]OR “multi strategic program*”[tiab] OR “multi level intervention*”[tiab] OR “multi level strateg*”[tiab] OR “multi level implementation*”[tiab] OR “multi level health promotion*”[tiab] OR “multi level program*”[tiab] OR “complex intervention*” [tiab] OR “complex strateg*”[tiab] OR “complex implementation*”[tiab] OR “complex health promotion*”[tiab] OR “complex initiative*”[tiab] OR “complex program*”[tiab] OR “health plan implementation*”[MeSH Terms] OR “health plan implementation*”[tiab] OR “preventive health service*”[MeSH Terms] OR “preventive health service*”[tiab]OR “health promotion*”[MeSH Terms] OR “health promotion*”[tiab] OR initiative*[tiab] OR program*[tiab] OR “multi component intervention*”[tiab] OR “multi component implementation*”[tiab] OR “multi component health promotion*”[tiab] OR “multi component initiative*”[tiab] OR “multi component program*”[tiab] OR intervention* [tiab]

#4 #1 AND #2 AND #3

LIMITERS initial search: 2013 – current; English language

LIMITERS updated search: 2023/08/02 – current; English language

Supplementary Material B: Overview Included Studies

| Reference | Country | Study Design | Population | Intervention Description | Key Findings |
| --- | --- | --- | --- | --- | --- |
| Positive Effects | | | | | |
| *Aldred et al. 2019 (1)* | UK, London | Natural | **General**  **Underrepresentation** of age 16-24, non-white, unemployed  **Age**: 16+ | “Mini-Holland program”: Improvements for walking and bicycling environments in three Outer London boroughs (Enfield, Waltham Forest, Kingston). **Bicycle infrastructure**: Physically protected cycle lanes. **Other infrastructure/environmental:** Cycle hubs at tube stations, measures to reduce and calm motor traffic, modal filtering (road closures to through motor traffic), transformation of road junctions into continuous footways (footway continued over the road to indicate pedestrian priority).  Construction = ni - planned completion in 2021/2022. June 2017: Third of components complete.  **BL**: May-June 2016. **FU:** May-June 2017.  **IG 1:** “mini-Holland”. Subdivided into 1.1 “High-dose mini-Holland”, substantial changes to local walking and bicycling infrastructure; 1.2 “Low-dose mini-Holland”, no substantial changes (yet). **CG:** “non-mini-Holland”, outer London borough not part of program. | Higher proportion of doing **cycling in past week** at follow-up in high-dose mini-Holland group compared to non-mini-Holland group (15.2% vs. 22.4%, 95% CI 1.02, 1.52, p=.04).  Higher **duration of past week AT** **(walking and cycling)** at follow-up in mini-Holland (low and high dose taken together) group compared to non-mini Holland group (23 extra minutes, 95% CI 10, 44.9, p=.04). Higher **duration of past week AT** at follow-up in high-dose mini-Holland group compared to non-mini-Holland group (41 extra minutes, 95% CI 7.0, 75.0, p=.02). |
| *Aldred et al. 2021 (2)* | UK, London | Natural | **General**  **Underrepresentation** of age 16-24, non-white, unemployed  **Age**: 16+ | “Mini-Holland program”: Improvements for walking and bicycling environments in three Outer London boroughs (Enfield, Waltham Forest, Kingston**). Bicycle infrastructure:** Physically protected cycle lanes. **Other infrastructure/environmental:** Cycle hubs at tube stations, measures to reduce and calm motor traffic, modal filtering (road closures to through motor traffic), transformation of road junctions into continuous footways (footway continued over the road to indicate pedestrian priority).  Construction = ni - planned completion in 2021/2022.  **BL**: May-June 2016. **FU 1:** May-June 2017. **FU 2:** May-June 2018. **FU 3:** May-June 2019  **IG 1:** “mini-Holland”. Subdivided into **1.1** “High-dose mini-Holland”, substantial changes to local walking and bicycling infrastructure; **1.2** “Low-dose mini-Holland”, no substantial changes (yet); **1.3** “proximity to routes”, <2km vs. 2-5km vs. >5km. **CG:** “non-mini-Holland”, outer London borough not part of program. | Higher proportion of doing **cycling in past week** at follow-up 1 and 3 in high-dose mini-Holland group compared to non-mini-Holland group (FU 1: point estimate 1.24, 95% CI 1.02, 1.52, p<.05; FU 3: point estimate 1.35, 95% CI 1.09, 167, p=.005), and at FU 3 for people living <2km compared to >5km from mini-Holland routes (point estimate 1.30, 95% CI 1.06, 1.61, p<.05). Increased duration of past week cycling at FU 3 in high-dose mini-Holland group compared to non-mini-Holland group (13.3 extra minutes, 95% CI 1.0, 25.6, p<.05), and for people living <2km compared to >5km from mini-Holland routes (10.6 extra minutes, 95% CI 0.7, 20.4, p<.05). Increased duration of **past week AT (walking and cycling)** at all three FUs in high-dose mini-Holland group compared to non-mini-Holland group (FU 1: 41 extra minutes, 95% CI 7.0, 75.0, p=.02; FU 2: 44min, 95% CI 10.5, 77.5; FU 3: 41.5min, 95% CI 3.3, 79.7), and at FU 2 and 3 for people living <2km compared to >5km from mini-Holland routes (FU 2: 35.7 extra minutes, 95% CI 6.6, 64.8, p<.05; FU 3: 33min, 95% CI 0.9, 65.1). |
| *Brown, Smith et al. 2016 (3)* | USA, Salt Lake City | Natural | **General**  **Age**: 18+ | **Bicycle** **infrastructure**: Improved high comfort bike lane (completion and widening of previous bike lane). **Other infrastructure/environmental**: Extension of light rail line, narrowed automotive lanes, better lid sidewalks.  Construction: ni - opening light rail in April 2013.  **BL**: March – December 2012. **FU**: May – November 2013.  **IG**: “near time 1” = residents at baseline living within 800m radius, and “near time 2” = follow-up within 800m radius. **CG**: “far time 1” = baseline within 801-2000m radius, and “far time 2” = follow-up within 801-2000m radius. | Intervention group participants at follow-up (IG “near time 2”) were more likely to take **transit-related** **active transportation trips** (all p≤.04), and to take **non-transit-related** **walk trips** along the intervention (all p<.001), compared to the other three groups They were also more likely to bicycle, but only compared to participants in the control group at baseline (CG “far time 1, 10%>5%, p=.04). |
| *Cook et al. 2016 (4)* | USA, Durham | Natural | **General**  **Age**: all ages (including children) | **Bicycle infrastructure**: Construction of bicycle and pedestrian bridge to link two segments of American Tobacco Trail (22mi shared use path).  Construction: between 2013-2014, no exact dates given.  **BL**: May-June 2013. **FU**: May 2014.  **IG**: Trail users. | 6% increase in **proportion of bicyclists** and 6% decrease in proportion of runners on trail (both p<.05). 4% increase in **trip duration** of all travel modes and 6% increase in trip duration of bicyclists (both p<.05).  133% increase in number of trips. 158% increase of proportion of children using trail. 27% increase in average trip distance (7.3 mi to 9.3 mi), with greatest increase for bicyclists (2.7 mi). Estimated total PA per week was 138min (not meeting PA guidelines) at BL, and 162min (meeting PA guidelines) at FU. |
| *Crane et al. 2017 (5)* | Australia, Sydney | Natural | **General**  **Age:** 18-55 | **Bicycle infrastructure:** New 2.4km bidirectional protected (separated from road and pedestrian traffic) cycleway. **Other infrastructure/environmental:** Pedestrian infrastructure improvements (footpaths, crossings, tree coverage), traffic calming measures (speed reduction, one-way traffic flow).  Construction = before October 2013 (no exact dates given) - opening cycleway in June 2014.  **BL**: October 2013. **FU 1**: September – October 2014 (post 4 months). **FU 2**: September-November 2015 (post 16 months).  **IG:** Suburbs surrounding cycleway (in analysis divided into 3 distance groups: <1km, 1.00-2.99km, >3km). **CG:** Suburbs without comparable bicycling infrastructure in similar distance to central business district (intervention destination). | **Living in the intervention area** associated with greater likelihood of **using cycleway** (OR:3.93, 95% CI=1.51,10.23, p<.0001). Strong correlation between **weekly bicycling and using the cycleway** (AOR=47.01, 95% CI= 8.66,255.19, p<.001) – correlation remained consistent between FU 1 – FU 2 (p=.03). IG showed higher **frequency of bicycling** than CG (p=.012). Increased **minutes of bicycling** seen in participants living at 1.00-2.99km, compared to <1km and >3km, distance from the cycleway (p=.007).  Non. sign. increased bicycling frequency seen in participants living at 1.00-2.99km, compared to <1km and >3km, distance from the cycleway (p=.08). 0.9%-6.4% increase in bicycle counts between BL-FU 2 – increase on cycleway in contrast to average decrease citywide. |
| *Frank et al. 2021 (6)* | Canada, Vancouver | Natural | **General**  **Age:** 18+ | **Bicycle infrastructure:** “Comox Greenway”. Retrofitted 2km route with cycling facilities (one-way shared on-street with counterflow lanes, one-way protected, two-way shared on-street, bicycle pavements markings for safer crossings). **Other infrastructure/environmental:** Restricted one-way travel for motor vehicles, intersection upgrades (signalization), street furniture (seating), green space, landscaping.  Construction: ni – June 2013.  **BL**: October 2012 - March 2013. **FU**: October 2014 - March 2015.  **IG**: Residents living within 300m radius of greenway. **CG**: Residents living further away than 300m of greenway. | Greenway exposure sign. associated with **cycling trip frequency**: IG increased their cycling trips by 252% compared to CG after opening of greenway (Model 4, IRR=3.52, 95% CI 1.54, 8.03, p=.003). |
| *Goodman, Panter et al. 2013 (7)* | UK | Natural, census data | **General**  **Age:** 16-74 | **Bicycle infrastructure:** New bicycle paths and greenways, improved bicycle paths. **Other infrastructure/environmental:** Bicycle parking stations, bicycle sharing stations, improved access to sharing stations. **Social-behavioral:** Lockers and showers at work, bicycle repairs, maintenance training, bicycle taster sessions, organized bicycle clubs and festivals, second-hand bicycle market.  Funding = October 2005 (CDTs), and April 2008 (CCTs), - March 2011.  **BL**: 2001. **FU:** 2011.  **IG**: 18 interventions towns (6 CDTs, 12 CCTs). **CG 1**: Matched comparison group (primary comparison group). **CG 2**: Unfunded comparison group. **CG 3:** Non-London national comparison group. | In intervention towns **cycling to work** (absolute effect size=0.69 (95% CI 0.66, 0.77); relative effect size=1.09, (95% CI 1.07, 1.11)), **walking to work** (effect size=1.7 (95% CI 1.62, 1.81)), and **taking public transport** (effect size=0.32 (95% CI 0.24, 0.41)) increased. **Driving to work** (effect size=-3.01 (95% CI -3.13, -2.88)) decreased. Equity impacts: Cycling, walking and driving to work effects were observed across different area deprivations with larger changes in more deprived areas. |
| *Goodman, Sahlqvist et al. 2013 (8)* | UK | Natural | **General**  **Age:** 18-89 | **Bicycle infrastructure:** Three “Connect2” projects: Traffic free bridges (in Cardiff and Kenilworth), Transformation of former informal riverside footpath into boardwalk (Southampton).  Construction: Cardiff July 2009 – July 2010, Kenilworth early 2009 – September 2011, Southampton April 2010 – July 2010.  **BL**: April 2010. **FU 1**: April 2011. **FU 2**: April 2012.  **IG:** Residents within 5km radius of intervention site. | 32% of participants **used intervention** site at FU1 and 38% at FU2 (r=.62). Most reported **mode of intervention site use** was walking for recreations (84% FU1, 85% FU2, r=.60), cycling for recreation (37% FU1, 39% FU2, r=.65), walking for transport (35% FU1, 32% FU2, r=.51), and cycling for transport (17% FU1, 18% FU2, r=.60). Strong **predictors of intervention site use** were living closer (e.g., FU2 adjusted RR=3.38 (95% CI 2.35, 4.87) for ≥4km vs. <1km distance), and higher BL walking and cycling levels (e.g., FU2 adjusted RR=2.09 (95% CI 1.55, 2.81) for >450 min/week vs. none). |
| *Goodman, Sahlqvist et al. 2014 (9)* | UK | Natural | **General**  **Age:** 18-89 | **Bicycle infrastructure:** Three “Connect2” projects: Traffic free bridges (in Cardiff and Kenilworth), Transformation of former informal riverside footpath into boardwalk (Southampton).  Construction: Cardiff July 2009 – July 2010, Kenilworth early 2009 – September 2011, Southampton April 2010 – July 2010.  **BL**: April 2010. **FU 1**: April 2011. **FU 2**: April 2012.  **IG:** Residents within 5km radius of intervention site. | 32% of participants **used intervention** site at FU1 and 38% at FU2. Most reported **mode of intervention site use** was walking for recreations (84% FU1, 85% FU2), cycling for recreation (37% FU1, 39% FU2), walking for transport (35% FU1, 32% FU2), and cycling for transport (16% FU1, 18% FU2). **Proximity to intervention:** Each kilometer closer to intervention site was associated with 15.3 minutes increase per week in walking and cycling (95% CI=6.5, 24.2), and an 12.5 minute increase per week in total PA (95% CI=1.9, 23.1). The effect was stronger in households without a car (46.8 min/week per km closer, 95% CI=21.6, 72.1) compared to households with a car (10.2 min/week, 95% CI=0.3, 20.1, interaction p=.007). |
| *Grunseit et al. 2019 (10)* | Australia, Sydney | Natural | **General**  **Age:** 18+ (survey), all ages (Ecounter) | **Bicycle infrastructure:** “Narrabeen Lagoon Trail” – 8.5 km multi-use recreational walking and cycling loop. Implementation of new closing segment (new bridges, 2km boardwalk). **Other infrastructure/environmental**: Reserve and car park upgrades, boat ramp, toilet facility upgrades, park furniture, rest stops, vantage outlook points, heritage restoration, environmental protection, planting vegetation.  Construction: 2010 (complete trail) / after November 2013 (new closing segment) – February 2015.  **BL** Ecounter: November 2012, Visual counts: October – December 2014. **FU 1** Ecounter: July 2015, Visual counts: March 2015. **FU 2** Visual counts: October – November 2015, survey: March + May 2015.  **IG:** Trail users. | Ecounter Level change pre- to post: Average increase in **bike counts** per week ranged from 1391 (95% CI 1107, 1675) to 1899 (95% CI 1672, 2126), and from 756 (95% CI 542, 970) -1149 (95% CI 9399, 1358) in **pedestrian counts**. Ecounter trend change pre- to post: Decrease in bike counts ranged from 50 (95% CI -73, -27) to -62 (95% CI -80, -44), and in pedestrian counts from -27 (95% CI -43, -10) to 8 (95% CI -42, 27). Mean bike and pedestrian counts were higher in 2015 compared to 2013 and 2014 (all p<.001). Increase in overall counts of users supported by visual count data. Users not meeting PA recommendations compared to users meeting PA recommendations were more likely to report increased **total PA** after loop completion (55.5% vs. 39.2%, p=.031) - 10.5 weeks post-completion (51.8% vs. 27.7%, p=.013), but not 3.5 weeks (60% vs. 56.3%, p=.742). |
| *Heesch et al. 2016 (11)* | Australia, Brisbane | Natural | **General**  **Age:** Adults | **Bicycle infrastructure:** “Veloway 1 Stage C”- segment of 17 km exclusive off-road bikeway.  Construction: ni – July 2013.  **BL** Field observations & survey: August 2009, GPS: January – June 2013. **FU** Field observations & survey: September 2013, GPS: July – December 2013.  **IG:** intervention site users. **CG 1:** SEFB (shared use off-road path) users. **CG 2:** Logan Road (main traffic road without bicycle infrastructure). | Higher average **bicycle trip distance** in IG (p=.02), and in commitment to making bicycle trips (p=.001) at FU compared to CG1. Monthly increase in **bicycle counts** in IG at FU (p<.002). compared to CG1 and 2. Monthly bicycle counts in CG1 and 2 did not change (p=.084). |
| *Heinen et al. 2015 (12)* | UK, Cambridge | Natural | **General**  **Age**: 16+ | “Cambridgeshire Guided Busway” – 25km separate off-road guided busway **(other infrastructure/environmental),** with separate walking and cycling path **(bicycle infrastructure).**  Construction: 2007 – 2011.  **BL:** 2009. **FU:** 2012.  **IG:** Intervention users (working in areas served by busway and living within 30km radius of city center). | Proximity (4 vs. 9km) to intervention was associated with increased likelihood of a large (>30%) increase (RRR=1.80, 95 % CI 1.27, 2.55), and decreased likelihood of a small (<30%) decrease (RRR=0.47, 95 % CI 0.28, 0.81) in share of **trips involving AT**. Also associated with increased likelihood of a large increase in share of trips made by car (RRR 2.09, 95 % CI 1.35, 3.21).  No association with changes in share of trips involving public transport, **number of commute trips**, and **commute distance**. |
| *Hirsch et al. 2017 (13)* | USA, Minneapolis | Natural, census data | **General**  **Age:** ni | **Bicycle infrastructure:** "Hiawatha Trail" and "Midtown Greenway" - 10.2 miles off-road paved paths, including bicycle and pedestrian bridge.  Construction: 2000 – 2007  **BL**: 2000. **FU**: 2010.  **IG**: Census tracts (n=116) users with different spatial exposure to new trails. | 2.3% increase in percentage of **workers commuting by bicycle** between BL and FU (p<.01). Negative association between **tract-level commuting by bicycle** and distance to the intervention sites (p<.01), and positive association between tract-level commuting by bicycle and proportion of commuting trips crossing the intervention sites (p<.01).  No differences in total number of work-related trips (p=0.97), or proportion of trips that potentially traverse the intervention sites (p=.95). |
| *Keall et al. 2015 (14)* | New Zealand, New Plymouth and Hastings | Natural, quasi-experimental | **General**  **Age:** 10+ (ACTIVE survey), all ages (NZTS survey) | "Let's Go" in New Plymouth and "iWay" in Hastings, together forming the "Model Communities Programme (MCP)". **Bicycle infrastructure:** New cycle lanes, cycle parking. **Other infrastructure/environmental**: Shared spaces with reduced speed limits for vehicles. **Social-behavioral**: AT media campaigns, events, cycle education.  Construction: 2011-2013, no exact dates given  **BL**: 2011. Mid-program: 2012. **FU**: 2013.  **IG**: Two MCP cities (New Plymouth, Hastings). **CG**: Two cities not part of funding (Whanganui, Masterton). | 37% increase in odds of **AT** **trips** (walking or cycling vs. other modes) in IG compared to CG from BL to FU (OR=1.37, 95% CI 1.08, 1.73). No differences in PA (walking or cycling) overall change over time. |
| *Law et al. 2014 (15)* | UK, London | Natural, model | **General**  **Age:** ni | **Bicycle infrastructure:** "Elephant and Castle" case study – Section 7 of "London Cycle Superhighway" (direct and continuos cycleway), general cycling landscape infrastructure (marked cycling lanes, segregated lanes), section 2 and 23 of "London Cycle Network" (segregated cycle tracks, cycle lanes, walking and cycling shared paths, **other infrastructure / environmental:** motor traffic speed reductions, rectification of potholes).  Construction: ni – 2011 (Superhighway).  **BL**: February 2003. **FU:** August 2012.  **IG:** Intervention site users. | **Increased average cyclist movement** between BL and FU (mean (SD)=35.714 (30.152) vs. 190.143 (126.801)). 55% in variation of cyclist movement at FU can be explained by distribution at BL (p<.01) - preference for more direct, continuous and safe routes at both time points. |
| *Panter et al. 2016 (16)* | UK, Cambridge | Natural | **General**  **Age:** 16+ | “Cambridgeshire Guided Busway” – 25km separate off-road guided busway **(other infrastructure/environmental),** with separate walking and cycling path **(bicycle infrastructure).**  Construction: 2007 – 2011.  **BL:** 2009. **FU:** 2012.  **IG:** Intervention users (working in areas served by busway and living within 30km radius of city center). | Intervention exposure associated with greater likelihood of increased **cycle commuting time** (RRR=1.34, 95% CI=1.03, 1.76, p<.05): Participants living 4km from busway 34% more likely to have increased cycle commuting time compared to residents living 9km away. Intervention exposure associated with increased **total cycling time** (RRR=1.32, 95% CI=1.04, 1.68, p<.05). Effect of intervention on active commuting moderated by BL active commuting (p=.02): Increased **total active commuting** **time** among participants with lowest BL total active commuting time (RRR=1.76, 95% CI=1.16, 2.67).  No sign. effect on total time spent in recreational or overall PA. |
| *Patterson et al. 2023 (17)* | UK | Natural | **General**  **Age:** 16+ | **Bicycle infrastructure**: New cycle paths (on and off-road). **Other infrastructure/environmental**: Traffic calming measures, speed restrictions, improved junctions, bicycle parking. **Social-behavioral:** Bicycle repairs, bicycle maintenance trainings, travel plans, bicycle breakfasts, bicycle festivals.  Funding: 2005 (CDTs) and 2008 (CCTs) – ni.  **BL**: 2001. **FU**: 2011.  **IG**: 18 interventions towns (6 CDTs, 12 CCTs). **CG 1**: Matched comparison group (primary comparison group). **CG 2**: Unfunded comparison group. **CG 3:** Participants living in areas successful in later 2013 funding scheme. **CG 4**: Non-London national non-intervention comparison group. | Increased **cycle commuting prevalence** in IG compared to CG at FU in women (AOR=1.56, 95% CI 1.16, 2.10). Increased **cycle commuting uptake** in IG compared to CG at FU in women (AOR=2.13, 95% CI 1.56, 2.91). |
| *Rissel et al. 2015 (18)* | Australia, Sydney | Natural | **General**  **Age:** 18-55 | **Bicycle infrastructure:** 2.4 km bi-directional separated bicycle path.  Construction: ni – June 2014.  **BL**: September – October 2013. **FU:** September – October 2014.  **IG:** Participants living within 2.5 km radius. **CG**: Participants living in comparison area in similar distance to city center, without new infrastructure. | 23%-97% increase in **bike counts** on intervention site, but **weekly cycling frequency** remained higher in the IG compared to CG (29.2-25.8% at FU vs. 22.4-23.2% at FU; p=.04), and did not change over time (p=.2). Higher proportion of participants in IG compared to CG **using the intervention** (24% vs. 7%; AOR= 3.58, 95% CI=2.01, 6.40, p=.001). Increased likelihood of **intervention use** with decreasing distance (500m increments) to intervention site (AOR=1.24, 95% CI=1.13, 1.37, p<.001). |
| *Shu et al. 2014 (19)* | USA, Santa Monica | Natural | **General**  **Age:** ni | **Bicycle infrastructure**: Retrofitted 1km segment of residential roadway: Widened sidewalks, more clear markings of crosswalks and bicycle lanes. **Other infrastructure/environmental**: E.g., raised center median with trees, pedestrian-scaled light poles, storm-water management.  Construction: December 2011 – February 2013.  **BL**: March-April 2011. **FU**: March-April 2013.  **IG**: Path users. | 37% increase in **pedestrian traffic volume** (except on weekday mornings, all p<.05).  No change in **cyclist traffic volume**. |
| *Skov-Petersen et al. 2017 (20)* | Denmark, Copenhagen | Natural | **General**  **Age:** ni | **Bicycle infrastructure**: Improvement of two routes: “Vestvolden” – 15km cycle greenway (new surface and light conditions), and “Albertslund” – 18km cycle highway.  Construction: ni – opening Vestvolden in October 2011 / opening Albertslund in April 2012.  **BL**: Survey: May 2011. **FU 1**: May 2012. **FU 2**: May 2013. Count data: October 2010 – September 2013.  **IG**: Intervention site users. **CG** (survey only): Control site (Roskildevej, not part of infrastructure improvements) users. | After improvement of greenway: Modest increases in **cyclist volume** on cycle highway (e.g., 7% increase on weekdays during day light rush hour (126 [95% CI 122, 130] - 135 [95% CI 129, 142] bicycles/hour). After improvement of cycle highway: Increased cyclist volume on both cycle greenway (e.g., 71% increase during weekday dark rush hours (14 [95% CI 8, 20] - 24 [95% CI 12, 37] bicycles/hour)), and highway (e.g., 61% increase during weekdays light rush hours (126 [95% CI 122, 130] – 203 [95% CI 195, 210] bicycles/hour)). Increased proportion of **new cyclists** between 2012 – 2013 (49-61% relocated from other routes, 4-6% induced cycling, both Fisher’s exact probability <.0001). |
| *Song et al. 2017 (21)* | UK | Natural | **General**  **Age:** 18+ | **Bicycle infrastructure:** Three “Connect2” projects: Traffic free bridges (in Cardiff and Kenilworth), Transformation of former informal riverside footpath into boardwalk (Southampton).  Construction: Cardiff July 2009 – July 2010, Kenilworth early 2009 – September 2011, Southampton April 2010 – July 2010.  **BL**: April 2010. **FU 1**: April 2011. **FU 2**: April 2012.  **IG:** Residents within 5km radius of intervention site. | Use of, but not proximity to infrastructure associated with shift towards AT at FU 1 (p=.05), and FU 2 (p=.01). 21-25% shift from driving to AT, but also 20-23% shift from AT to driving. Decreased walk time at FU 2(-13.30 min, p=.021). |
| No Effects | | | | | |
| *Aittasalo et al. 2019 (22)* | Finland, Tampere | Natural, randomized controlled | **General**  **Employees** of small – middle sized (n > 10) companies  **Age**: mean phase 1=43, mean phase 2 intervention group=46.7, mean phase 2 control group=41.1. | Phase 1: **Bicycle infrastructure:** Improvements on main and connecting walking and bicycling paths. Construction = 2014-2016.  **BL:** Fall 2014, spring 2015. **FU**: Fall 2016.  **IG**: Employees.  Phase 2: **Social-behavioral** work books for promoting ACW along with educational material.  **BL**: Fall 2016. **FU:** Spring 2017.  **IG**: Employees receiving social-behavioral strategies. **CG**: Employees, data collection only. Random allocation to experimental groups. | Phase 1: Change in self-reported mean **number of days with intention to bicycle at least part of work journey** in the following week (m=1.7 (SD=1.9) – m=1.9 (SD=1.7), p=.001).  No change in ACW.  Phase 2: Increased proportion of participants **willing to increase walking** (8,7%, 95% CI 1.8 to 15.6), and **bicycling** (5.5%, 95% CI 2.2 to 8.8)  Increased **proportion** of participants **having an opportunity to bicycle at least part of their work journey** (5.9%, 95% CI 2.1 to 9.7).  No change in **ACW**. |
| *Auchincloss et al. 2019 (23)* | USA, Philadelphia | Natural, census data | **General**  **Disadvantaged area**: poor, high crime rate, predominantly African-Amercian.   **Age**: ni | 1.5 mile urban greenway in southwest Philadelphia. **Bicycle infrastructure**: Retrofit of sidewalks and street segments into wide, tree-lined asphalt paved greenway. Included intersection improvements (sidewalk bump-outs, count down pedestrian signals, ADA ramps, improved markings). **Other infrastructure/environmental**: retrofit included new bust stop shelters, street trees, bicycle racks, signage, enhanced storm water management.  Construction: Winter 2012 – spring 2013.  **BL** (SOPARC, environmental audit): fall 2011. **FU** (SOPARC, environmental audit, survey): fall 2014.  **IG**: Intervention site users. **CG**: Comparison site (1-mile section at 3mi distance from intervention site) users. | 3%-7% increase in cycling, 4% to 9% increase in running or bicycling, 16% to 18% increase in MVPA (walking fast, running, bicycling) on intervention site.  No sign. difference in increase in MVPA over time between intervention and comparison sight (p >.15). |
| *Brown, Tharp et al. 2016 (24)* | USA, Salt Lake City | Natural | **General**  **Age**: 18+ | **Bicycle** **infrastructure**: Improved high comfort bike lane (completion and widening of previous bike lane). **Other infrastructure/environmental**: Extension of light rail line, narrowed automotive lanes, better lid sidewalks.  Construction: ni - opening light rail in April 2013.  **BL**: 2012. **FU**: May – November 2013.  **IG**: Participants living within 2km radius of intervention site; for comparison divided into groups: continuing cyclists (both 2012 and 2013), former cyclists (cycled in 2012 but not 2013), new cyclists (didn't cycle in 2012, but in 2013), never cyclists (bicycled in neither 2012 nor 2013). | Bicyclists (all three groups) compared to non-bicyclists showed higher **kcal expenditure** at baseline and follow-up (p<.001). Continuing cyclists had the highest kcal expenditure (2.67 kcal/minute more, p<.001). Higher kcal expenditure was shown for cyclists on cycling days vs. non-cycling days at baseline (4.82 kcal/min vs. 2.82 kcal/min, p=.0004) and follow-up (4.34 kcal/min vs. 2.96 kcal/min, p=.0009).  **Duration of bicycling** trips accounted for a sign. amount of kcal expenditure (.03 kcal/minute, p<.001). The intervention site accounted for 31%-41% of bicycling time of bicyclists. **BMI** levels did not differ across groups, but longer duration of bicycling trips was associated with a lower BMI (p=.03).  Non sign. increase in bicycling duration of continuing or former bicyclists from 18.51 min (SD=54.96) to 25.55 min (SD=49.95, p=.32). Complete cycling duration on the intervention site increased from 38.24 min (SD=71.01) to 43.92 min (SD=81.42, n.s.). |
| *Greaves et al. 2015 (25)* | Australia, Sydney | Natural | **General**  **Age:** 18-55 | **Bicycle infrastructure:** 2.4km separated bi-directional cycleway.  Construction: After November 2013 – June 2014.  **BL:** September – November 2013. **FU**: September – November 2014.  **IG**: Area surrounding intervention site. **CG**: Neighboring area without new cycleway. | No overall change in **cycle rates** (e.g., number of cycle trips in IG: 7.66% BL, 7.59% FU1, and CG: 4.48% BL, 4.20% FU1).  Non. sign. slight increase in cycle trip duration in IG (19.63 min BL, 23.12 min FU1). |
| *Heinen et al. 2017 (26)* | UK, Cambridge | Natural | **General**  **Age: 16+** | “Cambridgeshire Guided Busway” – 25km separate off-road guided busway **(other infrastructure/environmental),** with separate walking and cycling path **(bicycle infrastructure).**  Construction: 2007 – 2011.  **BL:** 2009, **FU 1**: 2010. **FU 2**: 2011. **FU 3** (post opening): 2012.  **IG:** Intervention users (working in areas served by busway and living within 30km radius of city center). | No association between exposure to intervention and modal shift (full or partial) or patterns of AT |
| *Nguyen et al. 2015 (27)* | Singapore, Tampine | Natural | **General**  **Age:** All ages | **Bicycle infrastructure:** Improved pedestrian and cycle paths (widening, demarcation, segregation) with distinction between small-scale upgrades (2.1 km) and area-wide upgrades (4.8 km).  Construction = Small-scale: 2009-2010; area-wide: 2011-2012.  **BL**: 2011. **FU:** 2013.  **IG**: Trail users, comparison of small-segments vs. area-wide vs. non-upgrade trails. | 44% increase in average **cyclist rate** on small-scale segments between BL and Fu (38.8 vs. 55.8 cyclists/hour, 93% CI, p=.07), in contrast to an 28% increase on wide-area segments (45 vs. 57.6, p=.21), and a 21% decrease on non-upgrade segments (26.4 vs. 20.8 cyclists/hour, p=.09).  Increased **cyclist volume** on 69% of small-scale segments compared to 50% of wide-area segments and 33% of non-upgrade segments. |
| *Ottoni et al. 2021 (28)* | Canada, Vancouver | Natural | **General**  **Age:** all ages | **Bicycle infrastructure**: “Arbutus Greenway” – 9km multi-modal transportation and recreation greenway. **Other infrastructure/environmental**: Benches, portable bathrooms.  Construction: 2016-2034. Temporary path construction phase (removal of original rail tiles, installment of paved path and mulch trail): 2017. Phase 1 construction (soil buffers, pavements markings demarcating lane use, benches, bathrooms): 2019.  **BL**: April-May 2017 (during temporary construction phase). **FU 1:** April-May 2018. **FU 2**: April-May 2019 (during Phase 1 constructions).  **IG:** Trail users. | 61% increase of greenway users from BL to FU2 (132 users/hour in 2017, 178 users/hour in 2018, 213 users/hour in 2019). Mostly related to cyclists: 90% increase (68/hour in 2017, 130/hour in 2019) in bicycle volume. Volume of pedestrians remained stable: 28% increase (57/hour in 2017, 58/hour in 2018, 73/hour in 2019). Mode split proportions in 2017: 52% cyclists, 43% walkers, 5% runners. Mode split proportions in 2019: 61% cyclists, 34% walkers, 5% runners. |
| *Stappers et al. 2021 (29)* | Netherlands, Maastricht | Natural | **General**  **Age:** 18+ | “The Green Carpet”: 2.3km path on top of double-layered tunnel (under previous highway). Two one-way streets separated by semi-paved middle section for cyclists, pedestrians and recreation (**bicycle infrastructure). Other infrastructure/environmental:** Greenery (grass, trees), benches, new lighting.  Construction: 2016 – spring 2018.  **BL**: September 2016 – June 2017. **FU**: September 2018 – June 2019.  **IG 1**:“High exposure“ group: Neighborhood directly bordering intervention site (East Maastricht). **IG 2**:”Low exposure” group: Neighborhood farther away (West-, North-West-, South-West Maastricht). **CG 1:”**No exposure” group: Heerlen residents. | No difference in **total or transport-related PA** changes over time between groups.  Within groups: Total PA: Percentage of MVPA decrease in IG 2 (B=-0.65, 95% CI -1.11, -0.20, p=.005). Transport-related PA: Percentage of MVPA decrease in CG 1 (B=-2.80,095% CI -5.00, -0.60, p=.13). PA at intervention site: Percentage of MVPA increase in IG 1 (B=8.80, 95% CI 1.18, 16.14, p=.024); percentage of LVP increase in IG 2 (B=34.01, 95% CI 16.16, 51.86, p<.001). |
| *Xiao et al. 2022 (30)* | France, Paris and Lyon | Natural | **General**  **Age:** ni | **Bicycle infrastructure**: New or improved cycle lanes (increased length, physical segregated cycle lanes, painted cycle lanes, shared lane markings).  Construction: Between 2014-2020 (different for each street).  **BL** and **FU**: Between January 2014 – March 2020 (six months pre- and post-implementation).  **IG 1**: 15 intervention streets. **CG 1:** 15 control streets (similar in pre-intervention cycle trends, >2km away from intervention streets). | No effect of cycling infrastructure on **cycle count** level or trend.  Non. significant: Increase in cycling counts per day in both IG and CG (Paris: 34.5%, 17.7%; Lyon: 24.5%, 8.2%). Greater pooled effect size for cycle level change in Paris (218 counts, 95% CI -189, 626, I^2^=0%) than in Lyon (34 counts, 95% CI -65, 133, I^2^=14%). |
| Negative Effects | | | | | |
| *Dill et al. 2014 (31)* | USA, Portland | Natural | **General**  **Adults with** min. 1 **child**  **Age:** Adults | **Bicycle infrastructure:** New bicycle boulevard (0.9-4.2mi).  Construction = between 2011 – 2012, no exact dates given.  **BL**: July-November 2010 and April-September 2011. **FU**: August-November 2012 and April – August 2013.  **IG**: Residents within 1000ft radius of boulevard street segments (8 street segments 0.9-4.2mi). **CG**: Residents within 1000ft radius of control street segments (11 street segments, parallel several blocks away, 1.0-5.7mi). | Negative correlation between being in treatment area after intervention installation and **minutes of bicycling** (if >10min, p=.00).  No effects for any other PA variable (MVPA per day, bicycling (>10min), walking (>20min), minutes of walking (if >20min), making bike trip, number of bike trips; all p>.05). |
| *Norwood et al. 2014 (32)* | Scotland | Natural | **General**  **Age:** 16+ | "Smarter Choices Smarter Places" (SCSP) program - **Bicycle infrastructure**: New cycleways, footpaths. **Other infrastructure/environmental:** Pedestrian crossings, cycle facilities, bus lanes, new bus services, bus shelters, ticketing improvements. **Social behavioral**: E.g., advertisements, social marketing, info material, promotional health walks.  Implementation: 2009-2012.  **BL**: May-June 2009. **FU**: May-June 2012.  **IG:** 7 SCSP areas**. CG:** 3 areas not part of SCSP program. | The proportion of participants meeting PA recommendations declined from BL to FU in both the IG (34.2% - 30.8%) and the CG (39.8% - 24.9%). There was a sign. difference between the groups at both time points (p< .01), with a less pronounced decline in the IG. |

Ni = no information. BL = Baseline. FU = Follow-up. IG = Intervention Group. CG = Control / Comparison Group. ACW = Active Commuting to Work. AT = Active Travel. BMI = Body Mass Index. PA = Physical Activity. CDT = Cycling Demonstration Towns. CCT = Cycling Cities and Towns. SEFB = South-East Freeway Bikeway. MVPA = Moderate to Vigorous Physical Activity. LPA = Light Physical Activity.

Supplementary Material C: Exemplary intervention components

| **Type** | | **Component** |
| --- | --- | --- |
| *Bicycle infrastructure* | | - On street cycle lanes with markings and/or physical protections - Off street cycle lanes segregated from traffic - Traffic free cycle and pedestrian bridges - Cycle highways - Greenways - Cycle boulevards - Bidirectional / contra flow cycle lanes - Shared use lanes for cycling and walking - Widening of lanes - More clear markings - Connection of trail segments - Advanced stop lines |
| *Other infrastructure/environmental* | for cycling | - Cycle parking - Cycle racks - Cycle hubs (stations to park and rent bicycles) - improved access to sharing stations - Rectification of potholes |
|  | for public transport | - Bus stop shelters - Public transportation signage - extended light rail line - new guided busway - improved bus network |
|  | for cars | - motor traffic calming measures:   - narrowed automotive lanes   - speed reductions   - one-way streets |
|  | for pedestrians | - Intersection improvements:   - Sidewalk bump-outs   - Countdown pedestrians’ signals   - Improved traffic signals - ADA ramps |
|  | for general public use | - multi-use recreational area   - toilet facilities   - boat ramp   - outlook points   - heritage restoration   - environmental protection - local vegetation - Street furniture - Street vegetation - Trash and recycling cans - Cycle trial – and playground - Better lighting - Signage - Enhanced storm water management |
| Social-behavioral | | - information center - lockers and showers at work - free personalized travel planning - cycling “taster” sessions - on-site cycle repairs - cycle maintenance trainings - cycle training - workbook with social-behavioral strategies to promote active travel to work - information material - cycle network - cycle user groups - “Bike breakfasts” - Annual workplace cycle challenges - Car and lift sharing - Bike officers - Second-hand bicycles - Cycle festivals - Advertisements - Promotional health walk |

# References

1. Aldred R, Croft J, Goodman A. Impacts of an active travel intervention with a cycling focus in a suburban context: One-year findings from an evaluation of London’s in-progress mini-Hollands programme. Transportation research part A: policy and practice. 2019;123:147-69.

2. Aldred R, Woodcock J, Goodman A. Major investment in active travel in Outer London: Impacts on travel behaviour, physical activity, and health. Journal of Transport & Health. 2021;20:100958.

3. Brown BB, Smith KR, Tharp D, Werner CM, Tribby CP, Miller HJ, et al. A complete street intervention for walking to transit, nontransit walking, and bicycling: a quasi-experimental demonstration of increased use. Journal of physical activity and health. 2016;13(11):1210-9.

4. Cook TJ, O’Brien SW, Jackson KN, Findley DJ, Searcy SE. Behavioral effects of completing a critical link in the american tobacco trail. Transportation Research Record. 2016;2598(1):19-26.

5. Crane M, Rissel C, Standen C, Ellison A, Ellison R, Wen LM, et al. Longitudinal evaluation of travel and health outcomes in relation to new bicycle infrastructure, Sydney, Australia. Journal of transport & health. 2017;6:386-95.

6. Frank LD, Hong A, Ngo VD. Build it and they will cycle: Causal evidence from the downtown Vancouver Comox Greenway. Transport Policy. 2021;105:1-11.

7. Goodman A, Panter J, Sharp SJ, Ogilvie D. Effectiveness and equity impacts of town-wide cycling initiatives in England: a longitudinal, controlled natural experimental study. Social science & medicine. 2013;97:228-37.

8. Goodman A, Sahlqvist S, Ogilvie D, Consortium i. Who uses new walking and cycling infrastructure and how? Longitudinal results from the UK iConnect study. Preventive medicine. 2013;57(5):518-24.

9. Goodman A, Sahlqvist S, Ogilvie D, Consortium i. New walking and cycling routes and increased physical activity: one-and 2-year findings from the UK iConnect study. American journal of public health. 2014;104(9):e38-e46.

10. Grunseit A, Crane M, Klarenaar P, Noyes J, Merom D. Closing the loop: short term impacts on physical activity of the completion of a loop trail in Sydney, Australia. International Journal of Behavioral Nutrition and Physical Activity. 2019;16(1):57.

11. Heesch KC, James B, Washington TL, Zuniga K, Burke M. Evaluation of the Veloway 1: A natural experiment of new bicycle infrastructure in Brisbane, Australia. Journal of Transport & Health. 2016;3(3):366-76.

12. Heinen E, Panter J, Mackett R, Ogilvie D. Changes in mode of travel to work: a natural experimental study of new transport infrastructure. International Journal of Behavioral Nutrition and Physical Activity. 2015;12(1):81.

13. Hirsch JA, Meyer KA, Peterson M, Zhang L, Rodriguez DA, Gordon-Larsen P. Municipal investment in off-road trails and changes in bicycle commuting in Minneapolis, Minnesota over 10 years: a longitudinal repeated cross-sectional study. International journal of behavioral nutrition and physical activity. 2017;14(1):21.

14. Keall M, Chapman R, Howden-Chapman P, Witten K, Abrahamse W, Woodward A. Increasing active travel: results of a quasi-experimental study of an intervention to encourage walking and cycling. J Epidemiol Community Health. 2015;69(12):1184-90.

15. Law S, Sakr FL, Martinez M. Measuring the changes in aggregate cycling patterns between 2003 and 2012 from a space syntax perspective. Behavioral Sciences. 2014;4(3):278-300.

16. Panter J, Heinen E, Mackett R, Ogilvie D. Impact of new transport infrastructure on walking, cycling, and physical activity. American journal of preventive medicine. 2016;50(2):e45-e53.

17. Patterson R, Ogilvie D, Laverty AA, Panter J. Equity impacts of cycling investment in England: A natural experimental study using longitudinally linked individual-level Census data. SSM - Population Health. 2023;23:101438.

18. Rissel C, Greaves S, Wen LM, Crane M, Standen C. Use of and short-term impacts of new cycling infrastructure in inner-Sydney, Australia: a quasi-experimental design. International Journal of Behavioral Nutrition and Physical Activity. 2015;12(1):129.

19. Shu S, Quiros DC, Wang R, Zhu Y. Changes of street use and on-road air quality before and after complete street retrofit: An exploratory case study in Santa Monica, California. Transportation Research Part D: Transport and Environment. 2014;32:387-96.

20. Skov-Petersen H, Jacobsen JB, Vedel SE, Alexander SNT, Rask S. Effects of upgrading to cycle highways-An analysis of demand induction, use patterns and satisfaction before and after. Journal of transport geography. 2017;64:203-10.

21. Song Y, Preston J, Ogilvie D, Consortium i. New walking and cycling infrastructure and modal shift in the UK: A quasi-experimental panel study. Transportation research part A: policy and practice. 2017;95:320-33.

22. Aittasalo M, Tiilikainen J, Tokola K, Suni J, Sievänen H, Vähä-Ypyä H, et al. Socio-ecological natural experiment with randomized controlled trial to promote active commuting to work: process evaluation, behavioral impacts, and changes in the use and quality of walking and cycling paths. International journal of environmental research and public health. 2019;16(9):1661.

23. Auchincloss AH, Michael YL, Kuder JF, Shi J, Khan S, Ballester LS. Changes in physical activity after building a greenway in a disadvantaged urban community: A natural experiment. Preventive medicine reports. 2019;15:100941.

24. Brown BB, Tharp D, Tribby CP, Smith KR, Miller HJ, Werner CM. Changes in bicycling over time associated with a new bike lane: relations with kilocalories energy expenditure and body mass index. Journal of transport & health. 2016;3(3):357-65.

25. Greaves S, Ellison R, Ellison A, Crane M, Rissel C, Standen C, editors. Changes in cycling following an infrastructure intervention. Australasian Transport Research Forum; 2015.

26. Heinen E, Harshfield A, Panter J, Mackett R, Ogilvie D. Does exposure to new transport infrastructure result in modal shifts? Patterns of change in commute mode choices in a four-year quasi-experimental cohort study. Journal of Transport & Health. 2017;6:396-410.

27. Nguyen PN, Koh PP, Wong YD, editors. Impacts of bicycle infrastructure: a case study in Singapore. Proceedings of the Institution of Civil Engineers-Municipal Engineer; 2015: Thomas Telford Ltd.

28. Ottoni CA, Sims-Gould J, Winters M. Safety perceptions of older adults on an urban greenway: Interplay of the social and built environment. Health & Place. 2021;70:102605.

29. Stappers NEH, Schipperijn J, Kremers SPJ, Bekker MPM, Jansen MWJ, de Vries NK, et al. Tunneling a crosstown highway: a natural experiment testing the longitudinal effect on physical activity and active transport. International Journal of Behavioral Nutrition and Physical Activity. 2021;18(1):111.

30. Xiao CS, Sharp SJ, van Sluijs EMF, Ogilvie D, Panter J. Impacts of new cycle infrastructure on cycling levels in two French cities: an interrupted time series analysis. International Journal of Behavioral Nutrition and Physical Activity. 2022;19(1):77.

31. Dill J, McNeil N, Broach J, Ma L. Bicycle boulevards and changes in physical activity and active transportation: Findings from a natural experiment. Preventive medicine. 2014;69:S74-S8.

32. Norwood P, Eberth B, Farrar S, Anable J, Ludbrook A. Active travel intervention and physical activity behaviour: An evaluation. Social science & medicine. 2014;113:50-8.
